# Exercise in patients with a tracheostomy and speaking valve: a randomised crossover-controlled trial

**DOI:** 10.1186/s13054-025-05621-2

**Published:** 2025-08-19

**Authors:** Luke Churchill, Lawrence Caruana, Nicole White, John F. Fraser, Allison Mandrusiak, Jennifer Paratz, Anna-Liisa Sutt, Peter J. Thomas, Stacey Verner-Wren, Oystein Tronstad

**Affiliations:** 1 Physiotherapy Department, Ground Floor, The Prince Charles Hospital, Chermside, QLD 4032 Australia; 2 Critical Care Research Group, The Prince Charles Hospital, Chermside, QLD Australia; 3 Australian Centre for Health Services Innovation, School of Public Health and Social Work, Queensland University of Technology, Brisbane, QLD Australia; 4 Institute for Molecular Bioscience, The University of Queensland, St Lucia, QLD Australia; 5 Intensive Care Unit, St Andrew’s War Memorial Hospital, Spring Hill, QLD Australia; 6 School of Rehabilitation and Health Sciences, The University of Queensland, St Lucia, QLD Australia; 7 School of Allied Health Sciences, Griffith University, Brisbane, Australia; 8 Swinburne University of Technology, Hawthorn, VIC Australia; 9 Speech and Language Therapy, The Royal London Hospital, London, E1 1FR UK; 10 Department of Physiotherapy, Royal Brisbane and Women’s Hospital, Herston, QLD Australia; 11 Department of Intensive Care, Royal Brisbane and Women’s Hospital, Herston, QLD Australia; 12 Department of Speech Pathology, The Prince Charles Hospital, Chermside, QLD Australia

Dear Editor,

Critically ill patients expected to receive prolonged invasive mechanical ventilation commonly receive a tracheostomy. Whilst a tracheostomy reduces sedation requirements and improves comfort, mobility, and communication, it is often associated with prolonged intensive care unit (ICU) and hospital length of stay [1]. These patients require exercise and rehabilitation which have well documented benefits in critical illness [2]. A speaking valve (SV) may be used to facilitate verbal communication and improve lung recruitment; however, is often removed during exercise due to potential (but unsubstantiated) safety concerns such as lung de-recruitment [3]. While the safety and benefits of SVs have been demonstrated with patients resting in bed, no studies have examined their impact during exercise. The aim of this study was to investigate the effect of performing exercise with a SV on lung function.

A randomised crossover-controlled study was performed in a large metropolitan, tertiary referral ICU. Ethical approval was obtained and written informed consent gained from all study participants.

Participants completed two 10-minute, in-bed cycle ergometry sessions in random order, either with a SV (intervention) or without a SV (control) in situ (Methods, online supplement). The primary outcome was safety, determined by changes in end-expiratory lung impedance (EELI) and tidal variation impedance (TVI) via electrical impedance tomography (EIT), and via monitoring of participants’ vital signs. Secondary outcomes included exercise performance measures and participant reported experiences of exercising with/without a SV. Adverse events were defined as reaching criteria that necessitated termination of the cycling session (Table E2).

Patients with a tracheostomy were screened according to inclusion/exclusion criteria (Table E1). Twenty eligible participants were recruited between May 2021 and June 2024 (median age 68 (54–71) years, 80% male) (Table E3). All participants completed both exercise sessions, and no adverse events occurred. Exercise performance measures were similar between control and intervention sessions with no statistically significant differences (Table E4). Lung aeration (EELI) reduced during exercise in both groups (Table E5), with significantly less lung aeration loss at mid-exercise and significantly larger increases in aeration during the recovery period when exercising with a SV (Table E7 and Fig. 1A). Across control and intervention sessions, TVI increased during exercise (Table E5), with significantly larger increases in the intervention group (Table E7 and Fig. 1B).

**Fig. 1:**
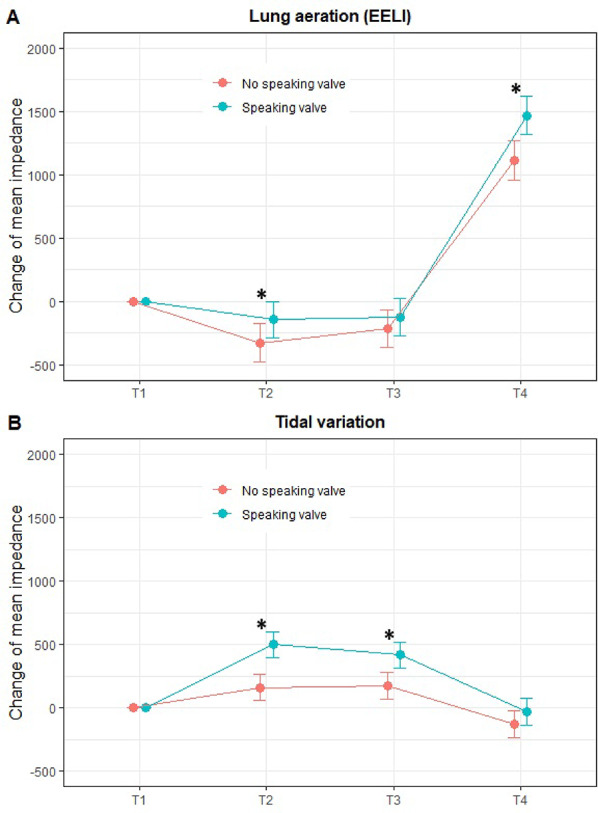
Magnitude of change in lung aeration (EELI) and tidal variation impedance (TVI) from baseline. Data are presented as estimate (95% CI) from linear mixed effects. **A**: No speaking valve vs. speaking valve (end-expiratory lung impedance (EELI)), **B**: No speaking valve vs. speaking valve (tidal variation). T1 = baseline, T2 = mid-exercise, T3 = end-exercise, and T4 = recovery (30 min post-exercise). *Values are statistically significant between control and intervention sessions. regression.

At baseline and exercise timepoints, peripheral oxygen saturation (SpO_2_) was significantly lower in the intervention group, although these differences were small and not clinically significant (Table E6). Mean oxygen saturations were maintained above 96% at all timepoints. Statistically significant differences in heart rate (HR) were observed at baseline and recovery, although these were also small and not clinically significant, with mean HR maintained below 100 bpm at all timepoints (Table E6). There were no significant differences in mean respiratory rate (RR) which was maintained below 26 across all timepoints (Table E6). Patient experience surveys (Appendix E2) completed by 18 participants demonstrated statistically significant differences in the ability to ask questions and make needs known when exercising with SVs (Figure E1 and Table E8), and 83% (*n* = 15) of participants preferred this intervention. Shortness of breath and satisfaction ratings were similar when exercising with or without a SV (Table E9).

The findings of this study indicate that exercise with a SV is safe and potentially beneficial for patients with a tracheostomy as highlighted by improvements in EELI and TVI. While there is currently no established minimally important difference in EELI or TVI (with impedance values expressed in arbitrary units) [4], these findings align with previous data demonstrating improved lung aeration associated with SV use for patients at rest [3], and would support a recommendation to keep a SV in situ for exercise to maximise lung recruitment and communication.

While some statistically significant differences were observed in vital signs, none were clinically significant or an indication of adverse events. Key clinical safety criteria for in-bed exercises with mechanically ventilated patients outline SpO_2_ levels ≥ 90%, RR ≤ 30, and ventricular rates < 120 bpm for any tachyarrhythmias [5], all of which were maintained in this study across both sessions at all timepoints. Exercise was as effective with or without a SV, with the added benefit of SVs allowing communication. While outside the scope of this study, the ability to communicate during exercise therapy in ICU may lead to improved engagement in rehabilitation and reduced feelings of anxiety and frustration.

The main study limitation was the choice of in-bed stationary cycling to allow the use of EIT. Early mobility and rehabilitation involve a range of interventions that may combine position changes and exercise (e.g. out-of-bed mobilisation). Therefore, the results may not be comparable to different types of upright exercise in patients with a tracheostomy, and this warrants further investigation.

In conclusion, our study indicates that exercise with a SV is safe and may be beneficial for improving lung aeration after exercise. It is also preferred by most patients compared to exercise without a SV. The findings of this study support patients with a tracheostomy and SV being able to safely engage in physical rehabilitation while maintaining autonomy in communication.

## Supplementary Information

Supplementary Material 1

## Data Availability

The datasets are available from the corresponding author on reasonable request.

## Abbreviations

Bpm: Beats per minute
EELI: End-expiratory lung impedance
EIT: Electrical impedance tomography
HR: Heart rate
ICU: Intensive care unit
RR: Respiratory rate
SpO_2_: Peripheral oxygen saturation
SV: Speaking valve
TVI: Tidal variation impedance

## Declarations

### Clinical Trial Number

The study was registered prior to commencement through the Australian and New Zealand Clinical Trials Registry (ANZCTR), Clinical Trial Number: 12619000148178.

## References

[1] Marella P, Ramanan M, Tabah A, Litton E, Edwards F, Laupland KB. Volume–outcome relationships for tracheostomies in Australia and New Zealand intensive care units: a registry-based retrospective study. Crit Care Resusc. 2025;27(1): 100096. 40109288 10.1016/j.ccrj.2024.12.002 PMC11919586

[2] Zhang L, Hu W, Cai Z, Liu J, Wu J, Deng Y, et al. Early mobilization of critically ill patients in the intensive care unit: a systematic review and meta-analysis. PLoS One. 2019;14(10):e0223185. 31581205 10.1371/journal.pone.0223185 PMC6776357

[3] Sutt A-L, Anstey CM, Caruana LR, Cornwell PL, Fraser JF. Ventilation distribution and lung recruitment with speaking valve use in tracheostomised patient weaning from mechanical ventilation in intensive care. J Crit Care. 2017;40:164–70. 28411422 10.1016/j.jcrc.2017.04.001

[4] Hickmann CE, Montecinos-Munoz NR, Castanares-Zapatero D, Arriagada-Garrido RS, Jeria-Blanco U, Gizzatullin T, et al. Acute effects of sitting out of bed and exercise on lung aeration and oxygenation in critically ill subjects. Respir Care. 2021;66(2):253–62. 32994357 10.4187/respcare.07487

[5] Hodgson CL, Stiller K, Needham DM, Tipping CJ, Harrold M, Baldwin CE, et al. Expert consensus and recommendations on safety criteria for active mobilization of mechanically ventilated critically ill adults. Crit Care. 2014;18:1–9. 10.1186/s13054-014-0658-y PMC4301888 25475522

