# Supplementary material for: Exercise in patients with a tracheostomy and speaking valve: a randomised crossover-controlled trial
Source: Crit Care. 2025 Aug 19;29:368. doi: 10.1186/s13054-025-05621-2 (PMC12366189; doi:10.1186/s13054-025-05621-2)
Supplement: Supplementary file 1 — Supplementary Material 1 [file 13054_2025_5621_MOESM1_ESM.docx]

**Electronic Supplement:**

**Exercise in patients with a tracheostomy and speaking valve: a randomised crossover-controlled trial.**

**Authors:**

Luke J. Churchill, BPhty^1,2^, Lawrence Caruana, BPhty^1,2^, Nicole White, PhD^2,3^, John F. Fraser, AO, MBChB, PhD, FRCP, FRCA, FFARCSI, FCICM^2,4,5^, Allison M. Mandrusiak, BPhty (Hons), PhD, GCHEd, SFHEA^6^, Jennifer Paratz, MPhty, FACP, GCMEd, MHMLaw, PhD^7,8^, Anna-Liisa Sutt, BSpPath, BA, MA, PhD^2,4,9^, Peter J. Thomas, BPhty (Hons), PhD, FACP^10,11^, Stacey Verner-Wren, BSpPath^12^, Oystein Tronstad, BPhty^1,2^.

**Affiliations:**

1. Physiotherapy Department, The Prince Charles Hospital, Chermside, QLD, Australia.
2. Critical Care Research Group, The Prince Charles Hospital, Chermside, QLD, Australia.
3. Australian Centre for Health Services Innovation, School of Public Health and Social Work, Queensland University of Technology, Brisbane, QLD, Australia.
4. Institute for Molecular Bioscience, The University of Queensland, St Lucia, QLD, Australia.
5. Intensive Care Unit, St Andrew’s War Memorial Hospital, Spring Hill, QLD, Australia.
6. School of Rehabilitation and Health Sciences, The University of Queensland, St Lucia, QLD, Australia.
7. School of Allied Health Sciences, Griffith University, Brisbane, Australia.
8. Swinburne University of Technology, Hawthorn, VIC, Australia
9. Speech and Language Therapy, The Royal London Hospital, London E1 1FR, United Kingdom.
10. Department of Physiotherapy, Royal Brisbane and Women’s Hospital, Herston, QLD, Australia.
11. Department of Intensive Care, Royal Brisbane and Women's Hospital, Herston, QLD, Australia.
12. Department of Speech Pathology, The Prince Charles Hospital, Chermside, QLD, Australia.

**Table of Contents**

[METHODS 2](#_Toc205819329)

[Study design 2](#_Toc205819330)

[Study participants 3](#_Toc205819331)

[**Table E1.** Inclusion / exclusion criteria 3](#_Toc205819332)

[Randomisation 3](#_Toc205819333)

[Measures 3](#_Toc205819334)

[**Table E2**. Cycling safety guidelines 4](#_Toc205819335)

[Exercise prescription 4](#_Toc205819336)

[Procedure 5](#_Toc205819337)

[Data analysis 5](#_Toc205819338)

[RESULTS 5](#_Toc205819339)

[Population description 5](#_Toc205819340)

[**Table E3.** Demographics and clinical characteristics 6](#_Toc205819341)

[Exercise performance measures 6](#_Toc205819342)

[**Table E4.** Comparison of exercise outcome measures, by intervention 7](#_Toc205819343)

[**Table E5.** Outcome measures across time periods compared with baseline 8](#_Toc205819344)

[**Table E6.** Comparison of outcome measures across time periods, by control or intervention 10](#_Toc205819345)

[Differences in lung aeration (EELI) and tidal variation 11](#_Toc205819346)

[**Table E7.** Magnitude of change in lung aeration and tidal variation from baseline, by control or intervention 12](#_Toc205819347)

[Subjective perception of exercise 13](#_Toc205819348)

[**Figure E1**. Participant experience survey responses (Q1-Q5) 13](#_Toc205819349)

[**Table E8.** Participant experience survey responses (Q1-Q5) 14](#_Toc205819350)

[**Table E9.** Participant experience survey responses (Q6 and Q7) 15](#_Toc205819351)

[REFERENCES 16](#_Toc205819352)

[APPENDICES 16](#_Toc205819353)

[Appendix E1 16](#_Toc205819354)

[Appendix E2 17](#_Toc205819355)

[Appendix E3 18](#_Toc205819356)

# METHODS

## Study design

This study used a randomised crossover-controlled design. The study was registered prior to commencement through the Australian and New Zealand Clinical Trials Registry (ANZCTR), Clinical Trial Number: 12619000148178. Human Research Ethics Committee approval was gained (HREC/18/QPCH/183) and the study was conducted in accordance with the ethical standards of the Declaration of Helsinki [1].

## Study participants

Patients with a tracheostomy were screened according to the inclusion and exclusion criteria (Table E1).

| **Table E1.** Inclusion / exclusion criteria |
| --- |
| 1. Inclusion:  - Age ≥ 18 years - Tracheostomy in situ - Able to use a SV as determined by the treating speech pathologist - Able to actively cycle for 10 minutes with a MOTOmed® Letto in-bed cycle ergometer as demonstrated in a prior physiotherapy session - Deemed to have capacity to provide informed consent and answer participant questionnaires |
| B. Exclusion:   - Injuries to lower limbs preventing participation in exercise - Rest in bed orders or bilateral non-weight bearing orders for lower limbs - Lower limb movement restrictions precluding exercise - Cardiovascular instability as determined clinically by the treating therapist or medical staff - Presence of femoral ECMO or IABP - Proven or suspected acute primary brain injury (e.g. traumatic brain injury, stroke, hypoxic brain injury) - Pre-existing cognitive impairment or language barrier that prohibits outcome assessments - Weight exceeding the safe working limit of the cycle ergometer (≥ 150 kg) - Death deemed imminent and inevitable |

ECMO: extracorporeal membrane oxygenation; IABP: intra-aortic balloon pump; ICU: intensive care unit; kg: kilograms; SV: speaking valve.

## Randomisation

Participants were randomised to complete the intervention or control session first using computer-generated software (https://www.randomizer.org). The participants served as their own control within the study, with crossover occurring after at least a two-hour washout period to allow participants to fully recover between the exercise sessions.

## Measures

The impact of the interventions on lung function was evaluated by electrical impedance tomography (EIT) using the PulmoVista 500 (Dräger, Lübeck, Germany) system. Lung aeration changes were measured using end-expiratory lung impedance (EELI) and tidal volume measured using tidal variation impedance (TVI). Data was recorded continuously throughout the entire data collection period from baseline to end of recovery, with a frame rate of 20 Hz and an operating frequency of 110 kHz. Data was analysed at the following timepoints: baseline (T1); mid-exercise (T2); end-exercise (T3); and 30 minutes after exercise (T4).

Vital signs throughout timepoints were measured via recording participants’ heart rate (HR) and peripheral oxygen saturation (SpO_2_) from the bedside monitoring system (Philips IntelliVue MX800) and respiratory rate (RR) from the participant’s ventilator (Maquet Servo-U® or HAMILTON-C6). Rating of perceived exertion (RPE) scores were measured using the modified BORG 0-10 scale [2] (Appendix E1). At T3, the cycling distance completed, watts generated, and average speed were obtained from the ergometer’s console. Adverse events were defined based on participants reaching criteria that would require the cycling session to be terminated (Table E2). Clinically significant changes were defined as SpO_2_ levels <90%, RR >30, and heart rates >120 bpm (for any tachyarrhythmias), based on previously published key clinical safety criteria for active mobilisation of patients receiving MV [3]. A difference in RPE was deemed clinically significant with a change of 1 point on the 10-point scale.

Participant experiences of exercising with or without a SV were collected immediately after the exercise intervention via a survey developed by authors (LCh and SVW) using clinical knowledge and patient feedback obtained from several years of treating patients with a tracheostomy (Appendix E2). Surveys comprised of five questions investigating participants’ ability to communicate, ask questions, make needs known, and participate in exercise (via a 5-point Likert scale). The survey also consisted of two questions using a 0-10 scale for shortness of breath (SOB) and overall satisfaction during exercise with or without the SV.

| **Table E2**. Cycling safety guidelines* |
| --- |
| 1. Cycling should not occur if any of the following conditions are present for greater than 15 minutes in the two hours prior to cycling:  - HR <50 or >140 bpm, or new arrhythmia - New onset of chest pain of potential cardiac origin - Mean arterial pressure <65 mmHg or less than the minimum set by medical consultant - Mean arterial pressure >120 mmHg or greater than the maximum set by medical consultant - Patients receiving vasoactive medications categorised in the ‘High’ category (Appendix E3) - Participant is pale / sweaty - FiO_2_ >0.8 - PEEP >15 cm H_2_O - SpO_2_ falls >10% of resting level or <85% for more than 60 seconds, or below target level for more than 60 seconds in participants with abnormal baseline SpO_2_ - Recent recipient of neuromuscular blocker medication - Clinical team’s opinion that participant should not receive cycling despite the absence of above criteria - Participant requests not to start due to feeling unwell |
| B. Criteria to terminate cycling session:   - HR increases further than the maximum exercise target HR, using the Karvonen heart rate method set at 70% [4] - New cardiac arrhythmia - Mean arterial pressure <65 mmHg or less than the minimum set by medical consultant - Mean arterial pressure >120 mmHg or greater than the maximum set by medical consultant - SpO_2_ falls >10% of resting level or <85% for more than 60 seconds, or below target level for more than 60 seconds in participants with abnormal baseline SpO_2_ - New onset of chest pain of potential cardiac origin - Participant is pale / sweaty - Participant reports excessive fatigue - Participant requests to stop due to feeling unwell - Patient declines to continue - Clinical team’s opinion that participant should stop cycling despite the absence of above criteria |

*developed from Heyland et.al. [5]

bpm: beats per minute; cm: centimetres; FiO_2_: fraction of inspired oxygen; HR: heart rate; H_2_O: water; mmHg: millimetres of mercury; PEEP: positive end-expiratory pressure; SpO_2_: peripheral oxygen saturation.

## Exercise prescription

Exercise was completed using an in-bed cycle ergometer (MOTOmed® Letto) for a maximum of 10 minutes. Participants were asked to exercise at a rate of perceived exertion (RPE) of 3-4 (“moderate” to “somewhat hard”) using the modified BORG scale [2] (Appendix E1). Individual resistance was set using the participant’s RPE following a one-minute trial of cycling prior to the data collection period. Resistance remained consistent throughout the exercise period; however, participants could alter their speed / rotation per minute (RPM) to maintain an RPE of 3-4.

## Procedure

Prior to the exercise sessions, participants were screened to ensure that they met safety criteria to perform cycling (Table E1). Participants were positioned in a semi-recumbent position in their bed with head of bed elevation set at 45 degrees. The EIT electrode belt was positioned around the participant’s thorax at the level of the fifth or sixth intercostal space. All measures were taken to maintain the position of participants throughout the entire recording period to minimise any changes in regional ventilation. Exercise was ceased at a maximum of 10 minutes, or if any pre-determined safety thresholds were met. After measurement of T4 data, the EIT thoracic belt was removed, then later reapplied and the baseline reset for the second exercise session.

## Data analysis

*Electrical impedance and physiological parameters*

Electrical impedance data were processed offline using commercially available Dräger software (Dräger PC Software Pulmovista 500, SW Version 1.30). Once baseline files were allocated (T1), two minutes of data were subsequently analysed at each of the remaining timepoints (T2-T4). Data for EELI and TVI were sampled at the T1-T4 timepoints to calculate values for the means and 95% confidence intervals (CI). Linear effects mixed regression analysis was used to compare differences between the control and intervention sessions over the T1-T4 timepoints for EELI, TVI, SpO_2_, RR, HR, and RPE; and to compare cycling distance, watts generated, average speed between the control and intervention at T3, and RPE. Models included fixed effects for SV or no SV, timepoints, and their two-way interaction. Post hoc pairwise comparisons were used to compare differences between control and intervention conditions for the same outcomes listed above. Model output was reported as least squares means (estimate with 95% CI), including differences between control and intervention sessions (estimate with 95% CI).

*Survey responses*

All available participant responses (numeric survey responses) were analysed, with missing values excluded. Fisher’s exact tests were used for responses using Likert scales and paired t-tests for SOB and overall satisfaction ratings. Preferences for exercise with or without a SV were reported as a percentage of total participants included in the analysis.

Statistical significance for all hypothesis tests was set at p ≤0.05. All statistical analyses were conducted using data program R (version 4.4.1; R Project for Statistical Computing; available at: https://www.r-project.org/).

# RESULTS

## Population description

Participant demographics and clinical characteristics are presented in Table E3. All participants were using a Passy-Muir^TM^ 007 SV [6]. There were 10 participants receiving high-flow oxygen therapy (HFOT) via a tracheostomy connection (Fisher and Paykel Optiflow Tracheostomy Interface) during both exercise sessions, eight participants on Pressure Support Ventilation (PSV), and two participants who received HFOT and PSV during separate assessment periods. All participants were still requiring intermittent MV during the day / night.

| **Table E3.** Demographics and clinical characteristics |  |  |
| --- | --- | --- |
| Subjects, n | 20 |  |
| Age, y | 68 (54 to 71) |  |
| Male | 16 (80%) |  |
| Height, cm | 176 (171 to 180) |  |
| Weight, kg | 90 (75 to 114) |  |
| BMI | 29 (26 to 36) |  |
| Primary reason for admission to ICU |  |  |
| Respiratory | 7 (35%) |  |
| Cardiac medical | 4 (20%) |  |
| Cardiac surgery | 4 (20%) |  |
| Thoracic surgery | 3 (15%) |  |
| Neurological | 1 (5%) |  |
| Trauma | 1 (5%) |  |
| ICU LOS total, days | 36 (24 to 52) |  |
| ICU LOS at recruitment, days | 27 (15 to 43) |  |
| Ventilation duration at recruitment, days | 26 (15 to 37) |  |
| Ventilation mode during exercise |  |  |
| HFOT | 10 (50%) |  |
| PSV | 8 (40%) |  |
| HFOT with SV & PSV without SV | 2 (10%) |  |

Data are presented as median (Q1 to Q3) or n (%)

Cm: centimetres; HFOT: high-flow oxygen therapy (>30 L/min of O_2_); ICU: intensive care unit; LOS: length of stay; Kg: kilograms; PSV: pressure support ventilation; SV: speaking valve.

## Exercise performance measures

Exercise performance measures were similar across control and intervention sessions with no statistically significant differences (Table E4). Adherence to the prescribed exercise intensity was observed with an increase in RPE values between T1 and T2 in both groups (Table E5) and modified Borg Scale ratings of three to four at T2 and T3 in both groups (Table E6). Between the control and intervention groups, the RPE was similar at T1 and T2 (Table E6). Statistically significant lower values for RPE were reported at T3 and T4 for the intervention group, but these were small (less than one) and not considered clinically significant.

| **Table E4.** Comparison of exercise outcome measures, by intervention | | | | |
| --- | --- | --- | --- | --- |
|  |  |  |  |  |
|  | SV | No SV | Difference | p-value |
| Active distance, Km | 1.6 (1.18 to 2.03) | 1.57 (1.15 to 2) | 0.0 (0.2 to -0.2) | 0.752 |
| Power, W | 2.72 (1.68 to 3.76) | 2.56 (1.52 to 3.6) | -0.2 (-0.5 to 0.1) | 0.288 |
| Peak power, W | 4.6 (3.26 to 5.94) | 4.4 (3.06 to 5.74) | -0.2 (-1.1 to 0.7) | 0.645 |
| Speed, RPM | 36.4 (30.5 to 42.2) | 37.4 (31.5 to 43.2) | 1 (-1.4 to 3.4) | 0.393 |

Results are presented as least squares means (95% CI) from linear mixed effects regression.

Km: kilometres; RPM: revolutions per minute; SV: speaking valve; W: watts.

| **Table E5.** Outcome measures across time periods compared with baseline | | | | | | | | | | |
| --- | --- | --- | --- | --- | --- | --- | --- | --- | --- | --- |
|  |  |  |  |  |  |  |  |  |  |  |
|  | Baseline (T1) | Mid-exercise (T2) | Difference* | p-value | End-exercise (T3) | Difference* | p-value | Recovery (T4) | Difference* | p-value |
| EELI (SV) | 848 (434 to 1262) | 702 (293 to 1111) | -146 (-293 to 1) | 0.053 | 724 (315 to 1132) | -124.3 (-271 to 23) | 0.130 | 2314 (1905 to 2724) | 1467 (1315 to 1618) | <0.001 |
| EELI (no SV) | 933 (519 to 1348) | 605 (196 to 1013) | -329 (-480 to -178) | <0.001 | 718 (310 to 1127) | -215 (-365 to -65) | 0.001 | 2045 (1636 to 2455) | 1112 (956 to 1268) | <0.001 |
| TVI (SV) | 1911 (1502 to 2321) | 2410 (2004 to 2817) | 499 (397 to 601) | <0.001 | 2331 (1924 to 2738) | 419 (317 to 522) | <0.001 | 1882 (1474 to 2289) | -30 (-135 to 75) | 0.885 |
| TVI (no SV) | 2149 (1740 to 2559) | 2309 (1902 to 2716) | 160 (55 to 265) | <0.001 | 2323 (1916 to 2730) | 174 (69 to 278) | <0.001 | 2022 (1614 to 2429) | -128 (-236 to -20) | 0.012 |
| SpO_2_ (SV) | 96.6 (95.6 to 97.7) | 96.2 (95.1 to 97.4) | -0.4 (-1.1 to 0.3) | 0.513 | 96.5 (95.4 to 97.7) | -0.1 (-0.9 to 0.7) | 0.986 | 97.4 (96.3 to 98.5) | 0.8 (0.1 to 1.4) | 0.014 |
| SpO_2_ (no SV) | 98.1 (97.1 to 99.2) | 97.9 (96.7 to 99) | -0.3 (-1.0 to 0.4) | 0.744 | 97.9 (96.7 to 99) | -0.3 (-1.1 to 0.5) | 0.814 | 97.4 (96.3 to 98.5) | -0.7 (-1.4 to -0.1) | 0.016 |
| RR (SV) | 24.1 (20.7 to 27.4) | 25.6 (22.2 to 29.1) | 1.6 (-0.6 to 3.8) | 0.248 | 25.2 (21.6 to 28.8) | 1.1 (-1.4 to 3.6) | 0.643 | 23.9 (20.5 to 27.3) | -0.2 (-2.1 to 1.7) | 0.995 |
| RR (no SV) | 23 (19.6 to 26.4) | 25.6 (22.1 to 29) | 2.5 (0.4 to 4.7) | 0.015 | 25 (21.4 to 28.5) | 1.9 (-0.6 to 4.4) | 0.194 | 23.7 (20.4 to 27.1) | 0.7 (-1.2 to 2.6) | 0.760 |
| HR (SV) | 93.8 (87.9 to 99.7) | 99.1 (93.1 to 105) | 5.3 (1.7 to 8.8) | <0.001 | 99.1 (92.9 to 105.3) | 5.3 (1.2 to 9.4) | 0.005 | 94.6 (88.8 to 100.5) | 0.8 (-2.3 to 4.0) | 0.901 |
| HR (no SV) | 91.2 (85.3 to 97) | 96.2 (90.2 to 102.1) | 5.0 (1.4 to 8.6) | 0.002 | 97.2 (91 to 103.4) | 6.1 (2.0 to 10.1) | <0.001 | 91.3 (85.4 to 97.2) | 0.2 (-3.0 to 3.3) | 0.999 |
| RPE (SV) | 0.65 (0.7 to 1.22) | 3.25 (2.8 to 3.7) | 2.6 (2 to 3.2) | <0.001 | 3.73 (3.2 to 4.2) | 3.1 (2.4 to 3.7) | <0.001 | 0.51 (-0.1 to 1.1) | -0.1 (-0.9 to 0.6) | 0.959 |
| RPE (no SV) | 0.5 (-0.1 to 1.1) | 3.33 (2.8 to 3.8) | 2.8 (2.2 to 3.4) | <0.001 | 4.23 (3.7 to 4.7) | 3.7 (3.1 to 4.4) | <0.001 | 1.07 (0.5 to 1.6) | 0.6 (-0.2 to 1.3) | 0.181 |

Results are presented as least squares means (95% CI) from linear mixed effects regression. *Difference calculated from reference point (T1).

EELI: end-expiratory lung impedance; HR: heart rate; RPE: rating of perceived exertion; RR: respiratory rate; SpO2: peripheral capillary oxygen saturation; SV: speaking valve; TVI: tidal variation impedance.

| **Table E6.** Comparison of outcome measures across time periods, by control or intervention | | | | | | | | | | | | | | | | |
| --- | --- | --- | --- | --- | --- | --- | --- | --- | --- | --- | --- | --- | --- | --- | --- | --- |
|  | Baseline (T1) | | | | Mid-exercise (T2) | | | | End-exercise (T3) | | | | Recovery (T4) | | | |
|  | SV | No SV | Difference | p-value | SV | No SV | Difference | p-value | SV | No SV | Difference | p-value | SV | No SV | Difference | p-value |
| SpO_2_ | 96.6  (95.6 to 97.7) | 98.1 (97.1 to 99.2) | 1.5 (1.0 to 2.0) | <0.001 | 96.2 (95.1 to 97.4) | 97.9 (96.7 to 99.0) | 1.6 (1.0 to 2.2) | <0.001 | 96.5 (95.4 to 97.7) | 97.9 (96.7 to 99.0) | 1.3 (0.6 to 2.1) | <0.001 | 97.4 (96.3 to 98.5) | 97.4 (96.3 to 98.5) | 0 (-0.5 to 0.5) | 0.992 |
| RR | 24.1 (20.7 to 27.4) | 23.0 (19.6 to 26.4) | -1.0 (-2.5 to 0.4) | 0.157 | 25.6 (22.2 to 29.1) | 25.6 (22.1 to 29.0) | -0.1 (-1.9 to 1.8) | 0.944 | 25.2 (21.6 to 28.8) | 25 (21.4 to 28.5) | -0.3 (-2.5 to 2.0) | 0.830 | 23.9 (20.5 to 27.3) | 23.7 (20.4 to 27.1) | -0.1 (-1.6 to 1.3) | 0.849 |
| HR | 93.8 (87.9 to 99.7) | 91.2 (85.3 to 97.0) | -2.7 (-5.0 to -0.3) | 0.027 | 99.1 (93.1 to 105.0) | 96.2 (90.2 to 102.1) | -2.9 (-5.9 to 0.1) | 0.061 | 99.1 (92.9 to 105.3) | 97.2 (91.0 to 103.4) | -1.9 (-5.6 to 1.8) | 0.316 | 94.6 (88.8 to 100.5) | 91.3 (85.4 to 97.2) | -3.3 (-5.7 to -0.9) | 0.007 |
| RPE | 0.65 (0.7 to 1.22) | 0.5 (-0.1 to 1.1) | -0.2 (-0.7 to 0.4) | 0.596 | 3.25 (2.8 to 3.7) | 3.33 (2.8 to 3.8) | -0.1 (-0.2 to 0.4) | 0.610 | 3.73 (3.2 to 4.2) | 4.23 (3.7 to 4.7) | 0.5 (0.1 to 0.9) | 0.013 | 0.51 (-0.1 to 1.1) | 1.07 (0.5 to 1.6) | 0.6 (0.0 to 1.1) | 0.049 |

Results are presented as least squares means (95% CI) from linear mixed effects regression.

HR: heart rate; RPE: rating of perceived exertion; RR: respiratory rate; SpO_2_: peripheral capillary oxygen saturation; SV: speaking valve.

## Differences in lung aeration (EELI) and tidal variation

Comparisons over time between baseline and each subsequent timepoint for control and intervention sessions separately are listed in Table E5. Comparisons in the magnitude of change from baseline between SV and no SV across each timepoint are presented in Table E7. Exercise with a SV demonstrated significantly less lung aeration loss at mid-exercise (T2) but not at T3. A significantly larger increase in aeration was observed in the intervention group during the recovery period (T4) (Table E7). Across both control and intervention sessions, TVI increased during exercise, with significantly larger increases observed in the intervention group (Table E7). At T4, the intervention group returned to its baseline level, but the control group was lower than its baseline (Table E5).

| **Table E7.** Magnitude of change in lung aeration and tidal variation from baseline, by control or intervention | | | | | | | | | | | | |
| --- | --- | --- | --- | --- | --- | --- | --- | --- | --- | --- | --- | --- |
|  | Mid-exercise (T2) | | | | End-exercise (T3) | | | | Recovery (T4) | | | |
|  | SV | No SV | Difference | p-value | SV | No SV | Difference | p-value | SV | No SV | Difference | p-value |
| EELI | -146 (-293 to 1) | -329 (-480 to -178) | -183 (-344 to -22) | 0.026 | -124 (-271 to 23) | -215 (-365 to -65) | -91 (-251 to 70) | 0.268 | 1467 (1315 to 1618) | 1112 (956 to 1268) | -354 (-520 to -189) | <0.001 |
| TVI | 499 (397 to 601) | 160 (55 to 264) | -339 (-451 to -228) | <0.001 | 419 (317 to 522) | 174 (69 to 278) | -246 (-358 to -134) | <0.001 | -30 (-135 to 75) | -128 (-236 to -20) | -98 (-213 to 17) | 0.094 |

Results are presented as least squares means (95% CI) from linear mixed effects regression.

EELI: end-expiratory lung impedance; SV: speaking valve; TVI: tidal variation impedance.

## Subjective perception of exercise

Overall, 18 participants completed the patient experience survey (Appendix E2). Surveys were missed by research staff in one participant, and a second participant declined due to fatigue. Of the questions compiled with a 5-point Likert scale (Q1-Q5, Appendix E2), two questions resulted in statistically significant differences: Q2) “I was able to ask questions during my exercise session”, and Q3) “I was able to make my needs known”, favouring the use of a SV (Table E8 and Figure E1). Similar responses were recorded across control and intervention sessions for SOB and satisfaction ratings (Table E9), however, 83% (*n* = 15) of participants preferred SVs, 6% (*n* = 1) preferred no SV, and 11% (*n* = 2) were neutral in their preference.

**Figure E1**. Participant experience survey responses (Q1-Q5).


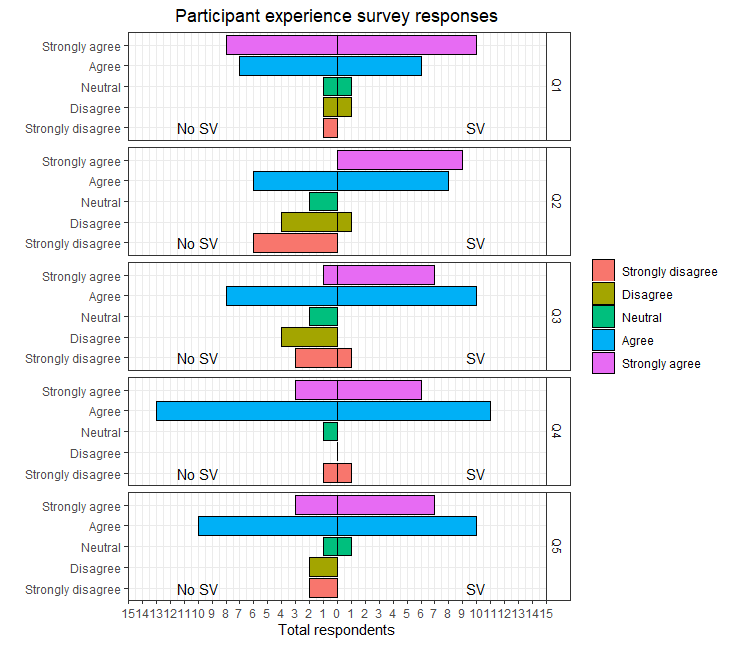


SV: speaking valve.

| **Table E8.** Participant experience survey responses (Q1-Q5) | | | | | | | | | | | |
| --- | --- | --- | --- | --- | --- | --- | --- | --- | --- | --- | --- |
|  |  |  |  |  |  |  |  |  |  |  |  |
|  | SV | | | | | No SV | | | | |  |
|  | % Strongly disagree (n) | % Disagree (n) | % Neutral (n) | % Agree (n) | % Strongly agree | % Strongly disagree (n) | % Disagree (n) | % Neutral (n) | % Agree (n) | % Strongly agree | p-value |
| Q1 - The ability to communicate during the exercise session was important to me | 0 (0) | 5.56 (1) | 5.56 (1) | 33.33 (6) | 55.56 (10) | 5.56 (1) | 5.56 (1) | 5.56 (1) | 38.89 (7) | 44.44 (8) | 0.927 |
| Q2 - I was able to ask questions during my exercise session | 0 (0) | 5.56 (1) | 0 (0) | 44.44 (8) | 50 (9) | 33.33 (6) | 22.22 (4) | 11.11 (2) | 33.33 (6) | 0 (0) | <0.001 |
| Q3 - I was able to make my needs known (such as pain, fatigue levels, other concerns) | 5.56 (1) | 0 (0) | 0 (0) | 55.56 (10) | 38.89 (7) | 16.67 (3) | 22.22 (4) | 11.11 (2) | 44.44 (8) | 5.56 (1) | 0.012 |
| Q4 - I was able to participate in my physio/exercise session | 5.56 (1) | 0 (0) | 0 (0) | 61.11 (11) | 33.33 (6) | 5.56 (1) | 0 (0) | 5.56 (1) | 72.22 (13) | 16.67 (3) | 0.711 |
| Q5 - My needs were listened to during my exercise session | 0 (0) | 0 (0) | 5.56 (1) | 55.56 (10) | 38.89 (7) | 11.11 (2) | 11.11 (2) | 5.56 (1) | 55.56 (10) | 16.67 (3) | 0.289 |

Data are presented as percentage (total participants)

SV: speaking valve

Values collected immediately after each exercise session.

| **Table E9.** Participant experience survey responses (Q6 and Q7) | | | |  |
| --- | --- | --- | --- | --- |
|  |  |  |  |  |
|  | SV | No SV | Difference | p-value |
| Q6 - Shortness of breath (Rating: /10) | 4.94 (3.72 to 6.17) | 4.67 (3.54 to 5.80) | 0.28 (-0.98 to 1.54) | 0.648 |
| Q7 - Satisfaction of exercise test (Rating: /10) | 8.61 (7.92 to 9.30) | 7.11 (5.76 to 8.46) | 1.5 (-0.16 to 3.16) | 0.073 |

Data are presented as mean (95% CI)

SV: speaking valve

Values collected immediately after each exercise session.

Rating: 0 = Nothing at all, 10 = Maximal.

# REFERENCES

1. Association WM. World Medical Association Declaration of Helsinki: ethical principles for medical research involving human subjects. Jama. 2013;310(20):2191-4.

2. Borg GA. Psychophysical bases of perceived exertion. Medicine & science in sports & exercise. 1982.

3. Hodgson CL, Stiller K, Needham DM, Tipping CJ, Harrold M, Baldwin CE, et al. Expert consensus and recommendations on safety criteria for active mobilization of mechanically ventilated critically ill adults. Critical care. 2014;18:1-9.

4. Karvonen MJJAMEBF. The effects of training on heart rate: A longitudinal study. 1957;35:307-15.

5. Heyland DK, Day A, Clarke GJ, Hough CT, Files DC, Mourtzakis M, et al. Nutrition and Exercise in Critical Illness Trial (NEXIS Trial): a protocol of a multicentred, randomised controlled trial of combined cycle ergometry and amino acid supplementation commenced early during critical illness. BMJ open. 2019;9(7):e027893.

6. Lian S, Teng L, Mao Z, Jiang H. Clinical utility and future direction of speaking valve: A review. Frontiers in Surgery. 2022;9:913147.

7. Boyd J, Paratz J, Tronstad O, Caruana L, Walsh J. Exercise is feasible in patients receiving vasoactive medication in a cardiac surgical intensive care unit: A prospective observational study. Australian Critical Care. 2020.

# APPENDICES

## Appendix E1

Modified BORG Scale

| 0 | Rest |
| --- | --- |
| 1 | Very easy |
| 2 | Somewhat easy |
| 3 | Moderate |
| 4 | Somewhat hard |
| 5 | Hard |
| 6 |  |
| 7 | Very hard |
| 8 |  |
| 9 |  |
| 10 | Very, very hard |

## Appendix E2

Participant Experience Survey

Instructor: Participant ID: SV during exercise: Yes / No

Please choose the answer that is closest to your experience during exercise for the following questions. These questions relate to the immediate exercise session you have just completed.

|  | Strongly Disagree | Disagree | Neither Agree Nor Disagree | Agree | Strongly Agree |  |
| --- | --- | --- | --- | --- | --- | --- |
| 1. The ability to communicate during the exercise session was important to me |  |  |  |  |  | |
| 1. I was able to ask questions during my exercise session |  |  |  |  |  | |
| 1. I was able to make my needs known (such as pain, fatigue levels, other concerns) |  |  |  |  |  | |
| 1. I was able to participate in my physio / exercise session |  |  |  |  |  | |
| 1. My needs were listened to during my exercise session |  |  |  |  |  | |

Please rate your shortness of breath during the exercise session?

0 1 2 3 4 5 6 7 8 9 10

Overall how satisfied were you with the exercise session?

0 1 2 3 4 5 6 7 8 9 10

Open-ended questions (To be asked following second intervention):

1. Any comments regarding exercising with or without a speaking valve?
2. Did you notice a difference in exercising with or without the speaking valve?
3. Which do you prefer?

## Appendix E3

Vasoactive medication Dose Classification [7]

**Table 1.** Vasoactive medication high dose category classification.

| **Category** | **Criteria** |
| --- | --- |
| High dose | - At least one vasoactive medication at a high dose. - Two vasoactive medications with at least one greater than low dose. - Greater than two vasoactive medications regardless of dose. |

**Table 2.** Classification of vasoactive medication high dosages.

| **Vasoactive medication** | **High (mcg/kg/min)** |
| --- | --- |
| Dopamine | >10 |
| Dobutamine | >10 |
| Adrenalin | >0.2 |
| Noradrenalin | >0.2 |
| Vasopressin | >0.03 |
| Levosimendan | >0.2 |
| Milrinone | >0.5 |
